# Combination Blockade of the IL6R/STAT-3 Axis with TIGIT and Its Impact on the Functional Activity of NK Cells against Prostate Cancer Cells

**DOI:** 10.1155/2022/1810804

**Published:** 2022-04-12

**Authors:** S. González-Ochoa, M. C. Tellez-Bañuelos, A. S. Méndez-Clemente, A. Bravo-Cuellar, G. Hernández Flores, L. A. Palafox-Mariscal, J. Haramati, E. J. Pedraza-Brindis, K. Sánchez-Reyes, P. C. Ortiz-Lazareno

**Affiliations:** ^1^Instituto Mexicano del Seguro Social (IMSS), Centro de Investigación Biomédica de Occidente (CIBO), División de Inmunología, Guadalajara, 44340, Jalisco, Mexico; ^2^Universidad de Guadalajara, Centro Universitario de Ciencias de la Salud (CUCS), Programa de Doctorado en Ciencias Biomédicas Orientación Inmunología, Guadalajara, 44340, Jalisco, Mexico; ^3^Universidad de Guadalajara, Centro Universitario de Ciencias Biológicas y Agropecuarias (CUCBA), Departamento de Biología Celular y Molecular, Las Agujas, 45220, Jalisco, Mexico; ^4^Universidad de Guadalajara, Centro Universitario de los Altos (CUALTOS), Departamento de Ciencias de la Salud, Tepatitlán de Morelos, 47620, Jalisco, Mexico; ^5^Universidad de Guadalajara, Centro Universitario de Ciencias de la Salud (CUCS), HIV and Immunodeficiencies Research Institute, Clinical Medicine Department, Guadalajara, 44280, Jalisco, Mexico; ^6^Universidad Autónoma de Guadalajara, Departamento Académico de Aparatos y Sistemas I, Unidad Académica de Ciencias de la Salud, Zapopan, 45129, Jalisco, Mexico

## Abstract

**Methods:**

We analyzed the secretion of cytokines, chemokines, and growth factors in 22Rv1, LNCaP, and DU145 cells. In these cells, we also evaluated the expression of NK ligands, IL6R, STAT-3, and phosporylated STAT-3. In NK-92 cells, we evaluated the effects of Stattic (Stt) and tocilizumab (Tcz) on NK receptors. In addition, we assessed if the disruption of the IL6R/STAT-3 pathway and blockade of TIGIT potentiated the cytotoxicity of NK-92 cells versus DU145 cells.

**Results:**

DU145 abundantly secretes M-CSF, VEGF, IL-6, CXCL8, and TGF-*β*. Furthermore, the expression of CD155 was found to increase in accordance with aggressiveness and metastatic status in the prostate cancer cells. Stt and Tcz induce a decrease in STAT-3 phosphorylation in the DU145 cells and, in turn, induce an increase of NKp46 and a decrease of TIGIT expression in NK-92 cells. Finally, the disruption of the IL6R/STAT-3 axis in prostate cancer cells and the blocking of TIGIT on NK-92 were observed to increase the cytotoxicity of NK-92 cells against DU145 cells through an increase in sFasL, granzyme A, granzyme B, and granulysin.

**Conclusions:**

Our results reveal that the combined use of inhibitors directed against the IL6R/STAT-3 axis and TIGIT enhances the functional activity of NK cells against castration-resistant prostate cancer cells.

## 1. Introduction

Prostate cancer (PCa) is the second most prevalent cancer (and the fifth most common cause of death in men) worldwide; in 2020, an estimation of 1.4 million cases and 375,000 deaths were reported globally [1]. These deaths are predominantly associated with resistance to treatments that ultimately lead to therapy failure and cancer progression. Among the causes associated with the failure of therapy against the tumor in PCa is a poor immune response. The predominant immune cells of the prostate microenvironment are T cells, B cells, and a small infiltrate of natural killer cells (NK) [2]; Since their discovery, it has been well known that NK cells play a decisive role in antitumor activity [3]. Specifically, in PCa, Marumo et al. were the first to describe the importance of the activity of NK cells against prostate cancer cells by observing a reduction in the functional capacity of NK cells in patients with metastatic prostate cancer compared to patients with localized tumors and healthy controls [4]. Other studies have reported that an increase in NK cell activity directly correlates with a better prognosis for patients and a lower risk of prostate cancer progression [2, 5]. Additionally, NK cells with high activity have been correlated with an increase in metastasis-free survival in patients with localized prostate cancer [6].

Moreover, it has been observed that the PCa microenvironment plays an essential role in regulating the activity of immune cells, including NK cells; this is mainly because the microenvironment is predominantly immunosuppressive [7]. The prostate cancer microenvironment has been reported to be characterized by a high secretion of regulatory molecules, such as TGF-*β*, IL-10, and IL-6 [8, 9]. Also, the presence of regulatory cells, such as Tregs, M2 macrophages, and MDSCs [10], as well as the expression of immunomodulatory ligands, such as PDL-1, and the secretion of potentially suppressive exosomes that contain MICA/B and ULBPs have been reported in the PCa mircroenvironment [11]. All these factors are determinants in the activity of NK cells against tumor cells.

In addition, it has been observed that STAT-3 is an important regulator of NK cell activity; some reports have demonstrated that STAT-3 signaling in tumor cells decreases the function of neighboring NK cells by inducing in tumor cells a lower expression of NK activating ligands (MICA/B and ULBPs) and chemoattractant factors (RANTES, IP-10, CCL2, CCL9, and CCL17). All of this leads to a decrease in the migration and activation of NK cells, with a corresponding decrease in cytotoxic mechanisms, and the downregulation of cytokine secretion (IFN-*γ* and TNF-*α*) and activating receptors (NKG2D and DNAM-1) [12].

Recently, poliovirus receptors (PVRs) have been identified as receptors present at an mRNA level in healthy tissues but with a low or absent protein expression, while in the tumor cells, this family of receptors (which also function as ligands) is overexpressed. These receptors are involved in processes such as tumor promotion and generation of metastasis; overexpression of these ligands contributes to immunological evasion through their interaction with T cell immunoglobulin and ITIM domain (TIGIT), an inhibitory receptor present on some NK and T cells [13–16].

Several studies have shown an overexpression of TIGIT in different types of cancer. This receptor is expressed predominantly on NK cells and plays an essential role in regulating their cytotoxic mechanisms in cancer, affecting NK cell proliferation and cytokine production, and directly disrupting their cytotoxicity activity; furthermore, TIGIT signaling has been associated with the generation of an exhausted condition on both CD8^+^ lymphocytes and NK cells [17–20]. Currently, around ten inhibitors directed against TIGIT have been developed; however, the utility of TIGIT as a target has not been verified completely, as these inhibitors are still being evaluated as possible therapeutic agents for different neoplasms in clinical trials from phases 1 to 3 [21].

In prostate cancer, high expression of PVR receptors (CD155 and CD112) has been reported in patients with resistant and castration-sensitive prostate cancer; for this reason, these receptors were proposed as potential targets in prostate cancer [22, 23]. Furthermore, a correlation between elevated expression of TIGIT and high-risk recurrence after radical prostatectomy has been shown [24]. However, no studies have currently reported using a strategy to combat prostate cancer using both blockade of TIGIT and the disruption of the IL6R/STAT-3 axis. Therefore, this study determines if the combined blockade of IL6R/STAT-3 and TIGIT enhances NK cell cytotoxicity against prostate cancer cells.

## 2. Material and Methods

### 2.1. Cell Culture Lines

The prostate cancer (PCa) cell lines DU145/ATCC® HTB-81™ (metastatic tumor and castration-resistant), LNCaP clone FGC/ATCC® CRL1740™ (metastatic tumor and androgen-dependent), and 22Rv1/ATCC® CRL2505™ (non-metastatic tumor) were obtained from the ATCC® (Manassas, VA, USA). All cell lines were cultured in RPMI 1640 medium (Life Technologies; Carlsbad, CA, USA) supplemented with 10% fetal bovine serum (FBS) (Gibco™, Thermo Fisher Scientific, Waltham, MA, USA) and 1% penicillin-streptomycin-neomycin (PSN) antibiotic mixture (Gibco™, Thermo Fisher Scientific Waltham, MA,USA). NK-92®/ATCC® CRL2407™ was cultured in a medium supplemented with 12.5% FBS together with 12.5% horse serum in addition to recombinant human IL-2 (PREPOTECH, Cranbury, NJ, USA) at 10 ng/mL concentration. Finally, RWPE1/ATCC® CRL11609™ (non-neoplastic prostate epithelial) was cultured with keratinocyte serum-free medium (KSFM) supplemented with 0.05 mg/mL bovine pituitary extract and 5 ng/mL EGF. The cells were kept in an environment of 95% air with 5% CO_2_ and a temperature of 37 °C. Adherent cells were treated with trypsin 0.25% (Gibco™, Thermo Fisher Scientific, Waltham, MA, USA) to detach the cells before passage or assay. The cell lines were cultured around 80-90% confluence to perform all the assays.

### 2.2. Antibodies and Reagents

Alexa Fluor® 647 antihuman TIGIT mAb (clone MBSA43) was obtained from BioLegend® (San Diego, CA, USA). The antibodies to evaluate CD155 (Alexa Fluor® 647, clone SKII.4), CD112 (PE, clone R2.525), PDL-1 (PerCP-Cy5.5, clone 29E.2A3), NKp30 (PE, clone P30-15), NKp44 (PE, clone P44-8), NKp46 (PE, clone 9E2), CD226 (PE, clone DX11), and NKG2D (PE, clone 1D11) were obtained from BioLegend® and MICA (PE, clone 159227), MICB (FITC, clone 236511), ULBP1 (PerCP, clone 170818), ULBP2-5-6 (PE, clone 165903), and B7-H6 (APC, clone 875002) from R&D Systems (Minneapolis, MN, USA). In addition, Ultra-LEAF ™ purified antihuman TIGIT antibody (clone A15153A, mouse IgG2a, BioLegend®) and anti-IL6R antibody (Tocilizumab/Actemra®; Roche, Basil, Switzerland) were used for functional blocking assays at 50 *μ*g/mL and 10 *μ*g/mL, respectively. Stattic (STAT-3 inhibitor; CAS 19983-44-9) was purchased from Santa Cruz Biotechnology (Santa Cruz, CA, USA). This reagent was reconstituted in dimethyl sulfoxide (DMSO, Sigma-Merck, Darmstadt, Germany) as 50 mM stock solutions and stored at −20 °C until use.

### 2.3. Quantification of Growth Factors and Cytokines in Prostate Cancer Supernatants

22Rv1, LNCaP, and DU145 prostate cancer cells (2 × 10^5^ cells) were seeded in 24-well plates to determine growth factors and cytokines after 12, 24, and 48 h. The supernatants were collected under sterile conditions and stored at −80 °C until the analysis. The expression of growth factors (LEGENDplex™ Human Growth Factor Panel, BioLegend®, San Diego, CA, USA) and cytokines (LEGENDplex™ Human Inflammation Panel, BioLegend®, San Diego, CA, USA) was determined through the LEGENDplex™ Multi-Analyte Flow Assay Kit, according to the manufacturer's protocol for each respective kit by flow cytometry. After the experiment, 2100 events from region Beads Size A were acquired in the Attune Acoustic Focusing Cytometer (Applied Biosystems®, Waltham, MA, USA). All data were analyzed using the BioLegend LEGENDplex™ version 8.0 Data Analysis Software (San Diego, CA, USA). The data are shown as the mean ± SD represented as pg/mL for each cytokine, chemokine, and growth factor.

### 2.4. Determination of NK Ligand Expression on Prostate Cancer Cells

22Rv1, LNCaP, and DU145 prostate cancer cells (1 × 10^5^ cells) were plated in 24-well plates allowing adhesion overnight before the experiment. After that,cells were harvested, two washes were carried out with PBS (Gibco™, Thermo Fisher Scientific, Waltham, MA, USA), and Zombie NIR ™ (BioLegend®, San Diego, CA, USA) was added and incubated for 20 min at room temperature to assess cell viability; next, after washing the cells, we added antibodies to evaluate all the ligands (CD155-Alexa Fluor® 647, CD112-PE, PDL-1-PerCP-Cy5.5, MICA-PE, MICB-FITC, ULBP1-PerCP, ULBP2-5-6-PE, and B7-H6-APC) and incubated for 30 minutes at room temperature protected from light. We washed the cells, and were resuspended in 500 *μ*L (PBS). Finally, 10,000 events from the viability region were acquired in the Attune Acoustic Focusing Cytometer (Applied Biosystems®, Waltham, MA, USA). The data were analyzed using Flowjo X 10.0.7r2 software (BD Biosciences, Franklin Lakes, NJ, USA). Data are shown as the mean ± SD of percentage expression and the median fluorescence intensity (MFI) for each evaluated ligand.

### 2.5. Metabolic Activity Assessment Assay

The effect of stattic on metabolism was determined using the WST-1 assay, a colorimetric technique based on the reduction of tetrazolium salts to formazan, which has been previously used to determine the metabolic activity of neoplastic and non-neoplastic prostatic cells [25–27]. For this, DU145 prostate cancer cells (2 × 10^4^ cells/well) and RWPE-1 (4 × 10^4^ cells/well) were seeded on a 96-well plate, allowing an overnight attachment. The medium was removed the next day, and the DU145 cells were treated with Stt (1, 3, 5, 7, and 10 *μ*M) and Tocilizumab (10 *μ*g/mL). RWPE-1 cells were treated or not with stattic (Stt), tocilizumab (Tcz), anti-TIGIT, Tcz + Stt, Tcz + anti-TIGIT, Stt + anti-TIGIT, and Stt + anti-TIGIT + Tcz) for 24 h. Additionally, etoposide (Et) was used as a positive control. Next, 2 h before the conclusion of the 24 h, we added 10 *μ*L of WST-1 reagent (Sigma-Aldrich, Co., Saint Louis, MO, USA) to each well. Finally, we obtained the absorbance at 480 nm wavelength with a correction reference wavelength of 650 nm using the microplate reader (Biotek Synergy HT, Winooski, VT, USA). All readings were normalized with the basal group considered as 100% metabolic activity.

### 2.6. Determination of Viability in DU145 Cells and NK Cells Treated with Stattic and Tocilizumab

NK-92 (1 × 10^5^ cells) and DU145 (3 × 10^4^ cells) were seeded in 24-well plates and treated with a concentration curve of stattic (1-10 *μ*M) and tocilizumab (10 *μ*g/mL) during 24 h. After cells were harvested, two washes were carried out with PBS (Gibco™, Thermo Fisher Scientific, Waltham, MA, USA), and the cells were stained with 7-AAD (BioLegend®, San Diego, CA, USA) for viability detection. Finally, 10,000 events were acquired through the Attune Acoustic Focusing Cytometer (Applied Biosystems®, Waltham, MA, USA). The data were analyzed with the Flowjo X 10.0.7r2 software (BD Biosciences, Franklin Lakes, NJ, USA). Data are shown as the mean ± SD represented as the viability percentage.

### 2.7. Evaluation of IL6R, STAT-3, and pSTAT-3 Expression on Prostate Cancer Cells by Western Blot

22Rv1 (3 × 10^6^ cells), LNCaP (1.5 × 10^6^ cells), and DU145 (1 × 10^6^ cells) were seeded in p100 Petri dishes, and 24 h after cell culture, we evaluated the expression of IL6R, STAT-3, and pSTAT-3 in the prostate cancer cells. In brief, the cells were harvested and lysed with 300 *μ*L of RIPA buffer (0.5% deoxycholate, 0.5% NP-40, 0.5% SDS, 50 mM Tris pH 8.0, and 150 mM NaCl) with a protease inhibitor cocktail (Roche Applied Science, Penzberg, DE, USA) and incubated on ice for 30 min. Next, the lysate was passed through a blunt 20-G needle fitted syringe 30 times and was sonicated (5 min, high level, 30 s on-off time interval) using the bioruptor sonicator (Diagenode, Liège, Belgium). The cells were incubated for 30 min, and then the solution was centrifuged (5 min at 12,000 rpm at 4°C) for protein collection; proteins were quantified with Bradford Assay Kit (Bio-Rad, Hercules, CA, USA), and primary electrophoresis was performed with polyacrylamide gels using 50 *μ*g of protein. Next, the proteins were transferred to a PVDF membrane (0.2 *μ*m) through a semidry system (BIORAD; Hercules, CA, USA), blocked for 2 h with 1× Western Blocking Reagent (LI-COR Biosciences, Lincoln, NE, USA) and incubated in agitation overnight with a primary antibody (1 : 1000) at 4°C. The following day, they were incubated with the immunofluorescence IRDye 800CW antigoat IgG secondary antibody (1 : 15000) for 2 h at room temperature. Membranes were washed and revealed using the Odyssey™ Infrared Imaging System (LI-COR Biotechnology, Lincoln, NE, USA). The primary antibodies used in the assays were STAT-3 (clone 124H6, Cell Signaling Technology Inc., MA, USA), IL6R*α* (clone H-7, Santa Cruz Biotechnology, CA, USA), pSTAT-3 (Tyr705, Cell Signaling Technology Inc., MA, USA), and *β*-actin (clone C4, Cell Signaling Technology Inc., MA, USA).

### 2.8. Dephosphorylation of STAT-3 by In-Cell Western (ICW)

The DU145 prostate cancer cells (3 × 10^4^ cells/well) were grown in a 96-well optical, black-walled transparent bottom plate (Thermo Fisher Scientific, Waltham, MA, USA). The cells were allowed to attach to the plastic and then were divided into an untreated group (basal) or treated with the following treatments: stattic (Stt), tocilizumab (Tcz), and Stt + Tcz for 24 h. The next day, cells were fixed using 100 *μ*L of methanol-acetone solution (3 : 1) for 20 min at −21 °C, and cell permeabilization was performed with 0.5% Triton X-100 for 5 min at room temperature twice; then, the cells were incubated at 4 °C overnight. The next day, the cells were blocked with LI-COR Odyssey blocking solution (LI-COR Biosciences, Lincoln, NE, USA) for 4 h and incubated with the primary mouse IgG antibodies against STAT-3 and pSTAT-3 (1 : 400 dilution, cell signaling) for 72 h. Finally, washes were performed and incubated with the goat antimouse IgG IRDyeTM 800 secondary antibody (dilution 1 : 15,000, LI-COR Biosciences, Lincoln, NE, USA) for 2 h at room temperature. The protein expression obtained through the integrated fluorescence intensities was detected using the Odyssey CLx Team ICW station (Odyssey Software Version 3.0, LI-COR Biosciences, Lincoln, NE, USA). The analysis of the relative expression of STAT-3 was performed by normalization with DRAQ5 staining (LI-COR Biosciences, Lincoln, NE, USA). Data are shown as the mean ± SD, represented as the percentage of STAT-3 phosphorylation.

### 2.9. Evaluation of Expression of NK Cell Receptors (NKG2D, NKp30, NKp44, NKp46, CD226, and TIGIT) by Flow Cytometry

NK-92 (1 × 10^5^ cells) were seeded in 24-well plates and were divided into an untreated group (basal) or treated with stattic (Stt), tocilizumab (Tcz), and Stt + Tcz for 24 h. After the cells were harvested, two washes were carried out with PBS (Gibco™, Thermo Fisher Scientific, Waltham, MA, USA); then, the antibodies against TIGIT-Alexa Fluor® 647, NKp30-PE, NKp44-PE, NKp46-PE, CD226-PE, and NKG2D-PE were placed and incubated for 30 minutes at room temperature protected from light. Next, the cells were washed, and then the cells were resuspended in 500 *μ*L (PBS). Finally, 7-AAD (BioLegend®, San Diego, CA, USA) was added. 10,000 events were acquired from the viability region through the Attune Acoustic Focusing Cytometer (Applied Biosystems®, Waltham, MA, USA). The data were analyzed with the Flowjo X 10.0.7r2 software (BD Biosciences, Franklin Lakes, NJ, USA). The data are shown as the mean ± SD of percentage expression and the median fluorescence intensity (MFI) for each evaluated NK receptor.

### 2.10. Real-Time NK Cell Cytotoxicity Monitoring in Coculture with Prostate Cancer Cells

DU145 target cells (3 × 10^4^ cells) were seeded in 96-well E-Plates (Agilent Technologies; Santa Clara, CA, USA) to assess the cell-mediated cytolytic activity by xCELLigence Real-Time Cell MP Analyzer (© 2021 Agilent Technologies Inc., Santa Clara, CA, USA). These cells were allowed to adhere to the plates for 19 hours with monitoring sweeps of 15 minutes; then, the medium was removed, and they were placed with the effector cells (NK-92) previously treated with anti-TIGIT in different ranges of the effector : target (5 : 1 and 10 : 1). After adding effector cells, all cocultures were treated according to the following groups [untreated group (basal), stattic (Stt), tocilizumab (Tcz), anti-TIGIT, Tcz + Stt, Tcz +anti-TIGIT, Stt + anti-TIGIT, and Stt + anti-TIGIT + Tcz). The data were recorded to determine the cell index for 24 hours using the immunotherapy platform of the xCELLigence RTCA software pro V2.3.4 (Agilent Technologies Inc., Santa Clara, CA, USA) [28]. Finally, the cytolysis percentage is determined using the following formula:

(1) $$\begin{array}{c} \%\text{Cytolisis}=\left(1–\frac{\text{Cell index at given E}:\text{T ratio}}{\text{Cell index without effector cells}}\right)\times 100 \end{array}$$

### 2.11. Quantification of Cytotoxicity Soluble Molecules from Coculture Supernatants

After finalizing the coculture experiments, the supernatant was collected at 4 and 24 h. The secretion of cytotoxicity molecules (IL-2, 4, 6, 10, 17A, IFN-*γ*, TNF-*α*, soluble Fas, soluble FasL, granzyme A, granzyme B, perforin, and granulysin) was determined using the human CD8/NK panel (13-plex) through the LEGENDplex™ Multi-Analyte Flow Assay Kit (BioLegend®, San Diego, CA, USA), according to the manufacturer's protocol for this kit by flow cytometry. After the experiment, 2100 events were acquired from region Beads Size A in the Attune Acoustic Focusing Cytometer (Applied Biosystems®, Waltham, MA, USA). All data were analyzed using the Biolegend LEGENDplex™ version 8.0 Data Analysis Software (San Diego, CA, USA). The data are shown as the mean ± SD represented as pg/mL for each cytotoxic molecule.

### 2.12. Statistical Analysis

Normality, homogeneity of variance, and data independence were determined before every analysis. The data analysis was carried out using descriptive statistics. Mixed effects two-way ANOVA was used to evaluate two variables and was performed with the Bonferroni test (post hoc) for cytotoxicity assays. In addition, parametric variables were analyzed with unpaired *t*-test and one-way ANOVA with the Tukey probe for comparison between two groups or Bonferroni for multiple comparison tests in all the other experiments. A statistically significant difference was considered when the *p* value was <0.05. GraphPad Prism V.8.1 software (San Diego, CA, USA) was used for all analyses.

## 3. Results

### 3.1. High IL-6, IL-8, and VEGF Secretion in DU145 Metastatic Castration-Resistant Prostate Cancer Cells

**Figure 1:**
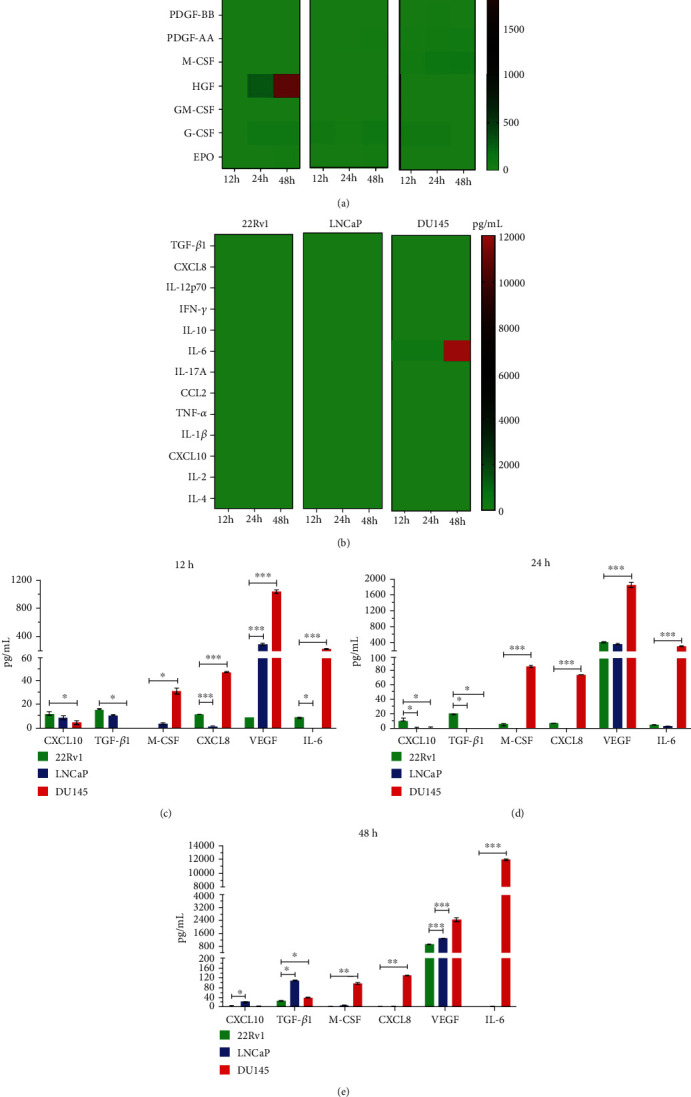
Advanced prostate cancer cells secrete a higher concentration of growth factors and cytokines according to stage progression. The cells were cultured at different times, the supernatants from PCa lines were collected, and 13 cytokines and 10 growth factors were evaluated using LEGENDplex™ technology. (a, b) The concentration of growth factors and cytokines from the 22Rv1, LNCaP, and DU145 supernatant was evaluated at 12, 24, and 48 h. (c,e) The concentration of soluble molecules increases, conforming with the advances of stage progression; the highest VEGF, M-CSF, CXCL8, and IL-6 values were observed in the DU145 cell line. Data are shown as the mean ± SD. ∗*p* < 0.05, ∗∗*p* < 0.01, ∗∗∗*p* < 0.001 (ANOVA with Tukey's multiple comparison test).

We assessed the secretion of growth factors, cytokines, and chemokines in 22Rv1, LNCaP, and DU145 cells at 12 h, 24 h, and 48 h (Figures 1(a) and 1(b), heat maps). We observed a higher secretion of IL-8, IL-6, M-CSF, and VEGF in DU145 prostate cancer cells when compared to 22Rv1 and LNCaP prostate cancer cells (∗∗∗*p* < 0.001). In contrast, CXCL10 (a chemokine involved in NK cell chemotaxis) was observed to be diminished in DU145 cells compared to the other prostate cancer cells (Figures 1(c) and 1(e), ∗*p* < 0.05). These results show that IL-8, IL-6, M-CSF, and VEGF were secreted proportionally according to the level of aggressiveness and metastatic capability reported for the prostate cancer cell lines and that this was most notably observed in the DU145 cell line.

### 3.2. DU145 Cells Have Increased Expression of CD155 (the Principal Ligand for TIGIT)

**Figure 2:**
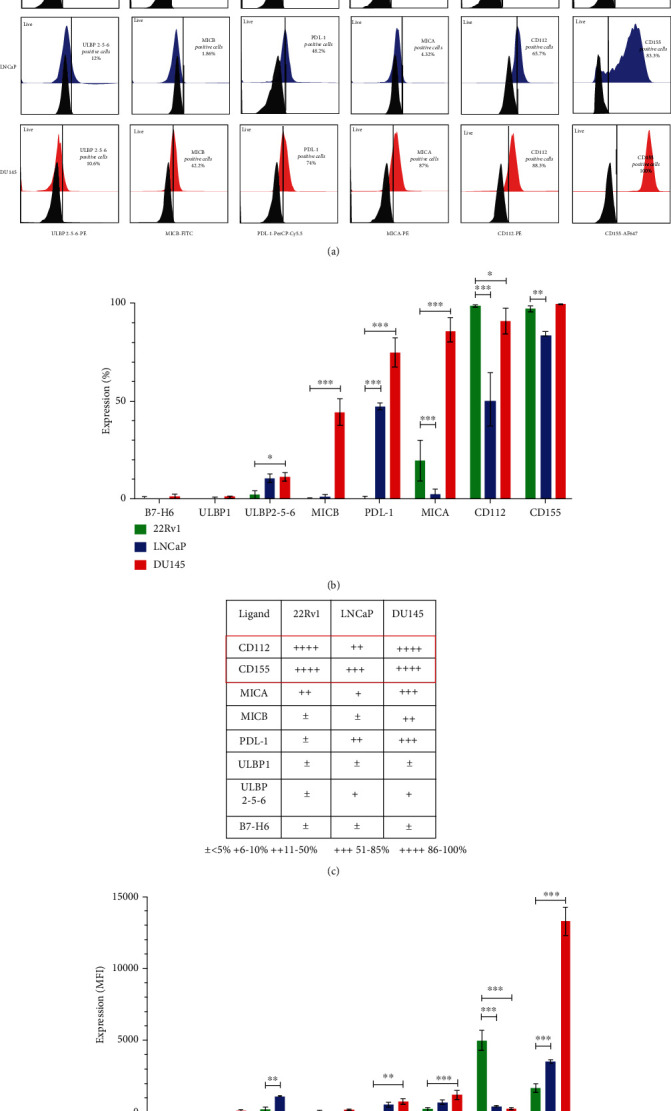
The expression of CD155 is increased in DU145 cells. The cells were cultured, and the expression of the ligands was subsequently evaluated by flow cytometry. (a) Ten thousand events from the region of the live cells were implemented for ligand evaluation. (b, c) The average percentage expression and (d) MFI of the ligands in the three PCa cell lines are shown. All experiments were repeated at least three times. Data are shown as the mean ± SD. ∗*p* < 0.05, ∗∗*p* < 0.01, ∗∗∗*p* < 0.001 (ANOVA with Tukey's multiple comparison test).

Since tumor cells are recognized by NK cells through an interplay of activating and inhibitory ligands, we decided to evaluate the expression of CD155, CD112, MICA, MICB, ULBP1-2-5-6, PDL-1, and B7-H6 on 22Rv1, LNCaP, and DU145 prostate cancer cells (Figure 2(a) and 2(d)). We observed that CD155 and CD112 were expressed at higher levels than MICA, MICB, ULBP1-2-5-6, PDL-1, and B7-H6 on all the prostate cancer cells (Figure 2(b) and 2(c), ∗∗∗*p* < 0.001). Also, when analyzing the MFI, we found a higher expression of CD155 compared to the other ligands in the DU145 cells; additionally, we observed that the expression of CD155 was very abundant in DU145 in comparison with the 22Rv1 and LNCaP prostate cancer cells (Figure 2(d), ∗∗∗*p* < 0.001). In contrast, 22Rv1 and LNCaP cells exhibited a higher expression of CD112 as compared with DU145 (Figure 2(d), ∗∗∗*p* < 0.001). These data indicate that the most aggressive cell line, Castration-resistant prostate cancer (CRPC) DU145 cells , exhibited the highest expression of CD155, which would be expected to interact with higher affinity with TIGIT, thus downregulating the activity of TIGIT^+^ NK cells. In contrast, the 22Rv1 and LNCaP cells exhibited lower expression of CD155 and higher expression of CD112, which has a greater affinity for the activating receptor CD226 (as shown in Figure 2(d)).

### 3.3. Stattic and Tocilizumab Decreased Phosphorylation of STAT-3 in Metastatic Castration-Resistant DU145 Prostate Cancer Cells

**Figure 3:**
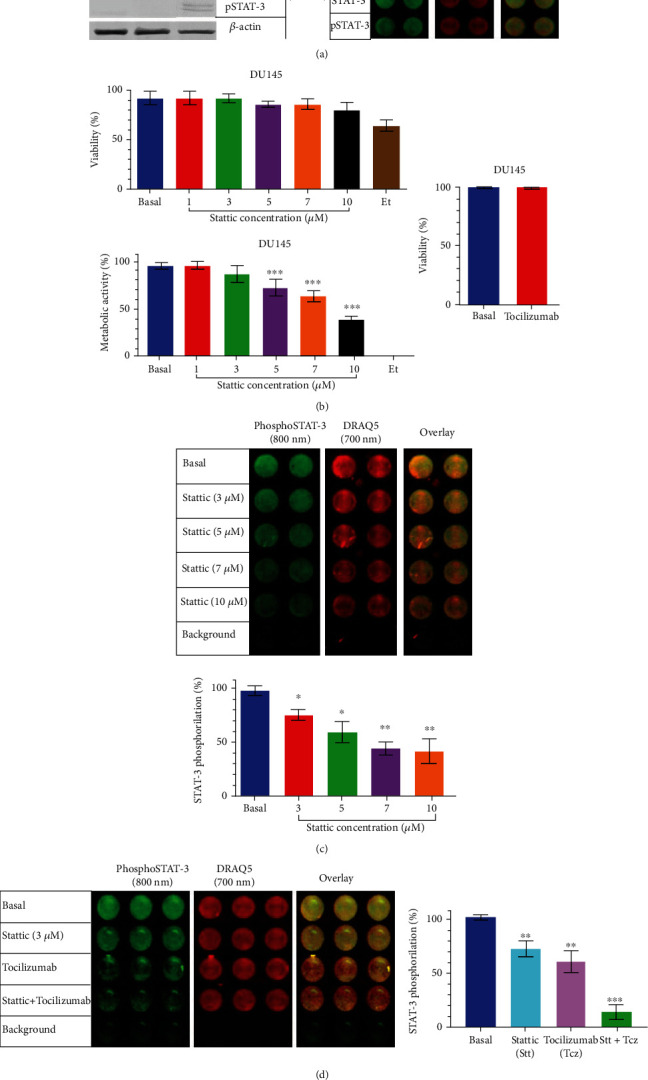
Stattic and tocilizumab combination increase pSTAT-3 dephosphorylation in DU145 cells. The cells were cultured with the different treatments according to the corresponding groups for 24 h. (a) IL6R/STAT-3 axis expression was compared between PCa cell lines using 50 *μ*g of protein by Western blot; the presence of constitutive pSTAT-3 expression was shown in the DU145 line only; protein expression was then verified by in-cell Western. (b) Use of treatment with Stt causes a decrease in metabolic activity with concentrations greater than 5 *μ*M. (c) The effective Stt decrease is only shown at concentrations above the IC50. (d) The combined treatments with Tcz allow a lower Stt concentration than the IC50 with greater efficiency in the dephosphorylation of pSTAT-3. All experiments were repeated at least three times. Data are shown as the mean ± SD. ∗*p* < 0.05, ∗∗*p* < 0.01, ∗∗∗*p* < 0.001; (unpaired *t*-test) against the basal group. Stt: stattic; Tcz: tocilizumab.

We first assessed the basal expression of the IL6R, STAT-3, and pSTAT-3 in 22Rv1, LNCaP, and DU145 prostate cancer cells. Interestingly, we observed that the DU145 cells exhibited a higher expression of the IL6R and a constitutive expression of pSTAT-3 when compared with the 22Rv1 and LNCaP prostate cancer cells (Figure 3(a)). Due to their high secretion of IL-6, IL6R, pSTAT-3 constitutive expression, and the high expression of the CD155 receptor, the main ligand of TIGIT, we decided to work with the DU145 cells in the following assays. First, we evaluated the effects of stattic (Stt) and tocilizumab (Tcz) on the viability and metabolic activity of the DU145 cells. We did not observe significant changes with Stt and Tcz in viability (Figure 3(b)); however, Stt concentrations higher than 5 *μ*M decreased metabolic activity in DU145 cells (Figure 3(b), ∗∗∗*p* < 0.001). Additionally, we compared the effects of treatments on non-neoplastic RWPE-1 cells, without observing any significant change in viability and metabolic activity (Figures S1(a) and S1(b)). Therefore, we evaluated the inhibition of phosphorylation with increasing concentrations of Stt, and we observed a significant decrease with concentrations starting from 1 *μ*M (Figure 3(c), ∗∗*p* < 0.01). Next, we evaluated the effect of Stt in combination with Tcz over STAT-3 phosphorylation in DU145 cells. To perform this experiment, the cells were treated with 10 *μ*g of tocilizumab [29], and, as we had observed that concentrations of 5 *μ*M of Stt reduced the metabolic activity in DU145 cells, we decided to work with a concentration of 3 *μ*M in the following tests [30]. We observed that the combination of Stt (3 *μ*M) + Tcz (10 *μ*g/mL) significantly decreased the phosphorylation of STAT-3 compared to the individual treatments (Stt and Tcz alone) (Figure 3(d), ∗∗∗*p* < 0.001). Our results indicate that the combination of Stt + Tcz effectively and synergistically inhibits the IL-6/IL6R/STAT-3 pathway in tumor cells.

### 3.4. Combined Treatment with Stattic and Tocilizumab Decreases the Expression of TIGIT and Increases the Expression of NKp46 in NK Cells

**Figure 4:**
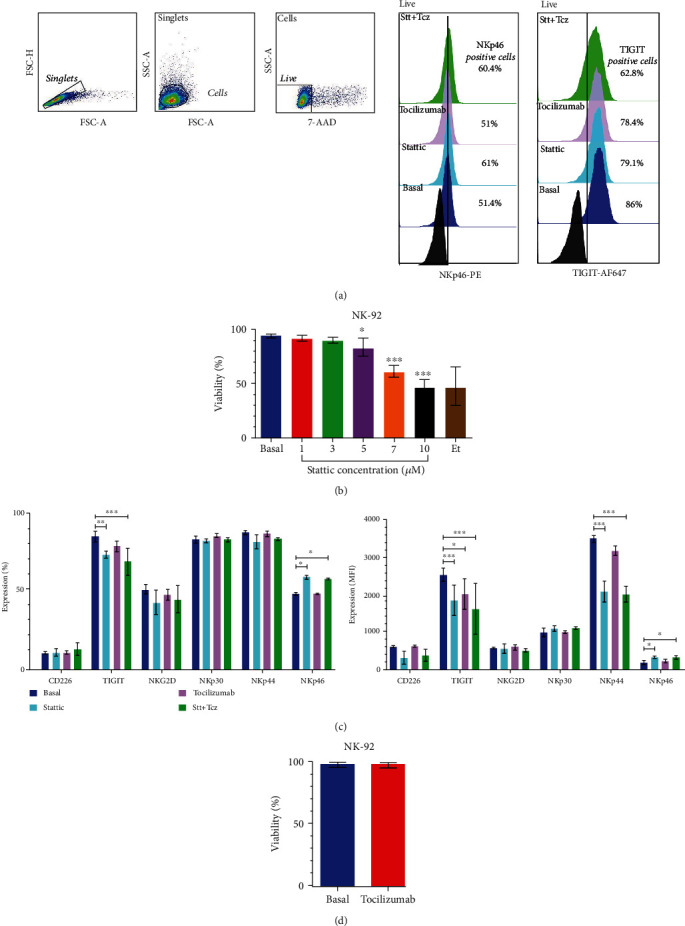
The combined use of stattic and tocilizumab increases the expression of NKp46 and decreases the expression of TIGIT in NK-92 cells. The NK-92 cells were cultured and treated with Stt at 3 *μ*M and 10 *μ*g/mL of Tcz for 24 hours. (a) Analysis strategy was used for evaluating the effectiveness of treatments on viability and receptor expression. (b) Use of Stt above 5 *μ*M causes a significant decrease in the viability of NK-92 cells. The data are shown as the mean ± SD. ∗*p* < 0.05, ∗∗*p* < 0.01, ∗∗∗*p* < 0.001; (unpaired *t*-test) against the basal group. (c, d) Treated groups with Stt and Tcz show a decrease in TIGIT and an increase in NKp46 in the percentage of positive cells and MFI; additionally, a significant decrease in NKp44 was only observed in the MFI. Data are shown as the mean ± SD. ∗*p* < 0.05, ∗∗*p* < 0.01, ∗∗∗*p* < 0.001 (ANOVA with Bonferroni multiple comparison test). All experiments were repeated at least three times. Stt: stattic; Tcz: tocilizumab.

We evaluated the effects of exposure to Stt and Tcz on NK cells (Figure 4(a)). First, we observed that Tcz did not affect viability in the NK-92 cells, whereas Stt at concentrations of 5 *μ*M significantly decreased viability (Figure 4(b), ∗∗∗*p* < 0.001). Next, we tested the effect of the combined treatment over the expression of NKG2D, NKp30, NKp44, NKp46, CD226, and TIGIT receptors in NK cells. Interestingly, Stt + Tcz decreased the expression and MFI of TIGIT and increased NKp46 expression (Figures 4(c) and 4(d), ∗∗∗*p* < 0.001). These results show that treatment with Stt + Tcz can regulate the expression of receptors involved both in the activation and regulation of the cytotoxic activity of NK cells.

### 3.5. Stattic plus Tocilizumab and Anti-TIGIT Increases the Cytotoxicity of NK-92 Cells against DU145 Prostate Cancer Cells

**Figure 5:**
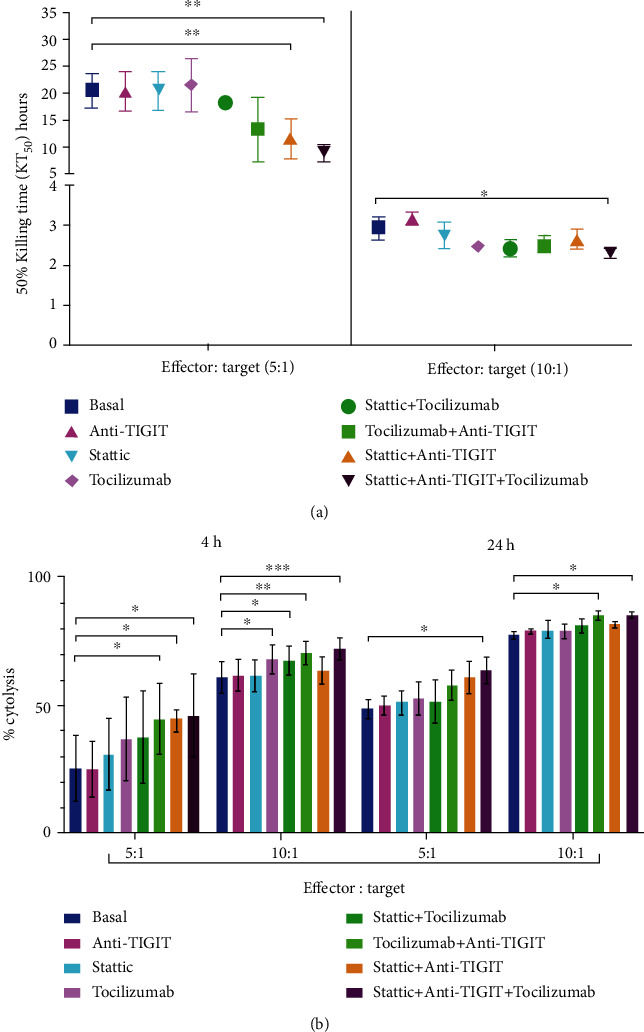
Stattic + Anti-TIGIT + Tocilizumab increase the cytotoxicity of NK-92 cells against DU145 prostate cancer cells. (a) Significant decrease in KT50 in the Stt + Anti-TIGIT + Tcz treated groups compared to the basal group. (b) A significant increase was observed in the percentage of cytolysis in the groups treated with Stt + Anti-TIGIT + Tcz compared with the basal group observed at 4 and 24 h of coculture in both ranges E : T. All experiments were repeated at least three times. Data are shown as the mean ± SD. ∗*p* < 0.05, ∗∗*p* < 0.01, ∗∗∗*p* < 0.001 (ANOVA with Bonferroni multiple comparison test). Stt: stattic; Tcz: tocilizumab.

The percentage of cytolysis at 4 h (short time activity) and 24 h (long time activity) was determined in NK-92 cells in coculture with DU145 cells treated or not with Stt and/or Tcz and/or anti-TIGIT using the effector : target ranges (E : T) 5 : 1 (Figure S2(a) and S2(b)) and 10 : 1 (Figures S2(c) and S2(d)). Interestingly, we observed a significant decrease in killing time 50 (KT50) in cocultures treated with Stt + Tcz and anti-TIGIT compared to other treatments in both E : T ranges; in particular, at 4 h, a decrease in KT50 was shown in cocultures with Stt + Tcz and anti-TIGIT (Figures 5(a) and 5(b), ∗∗∗*p* < 0.001). Likewise, we observed an increase in the cytotoxicity at 4 h in the cocultures treated with all treatments (Stt + Tcz + anti-TIGIT) compared to the basal group, and it was also observed that the three combined treatments maintained a significantly increasing cytotoxicity at 24 h of coculture in both ranges of E : T (Figures 5(c) and 5(d), ∗*p* < 0.05). Although, in general, treatment with either Stt, Tcz, or anti-TIGIT alone increased the cytotoxicity of NK-92 cells against DU145 cells, it is essential to note that the group with the simultaneous treatment with Stt, Tcz, and anti-TIGIT continued to maintain a high activity at 24 h, which correlated to higher cytotoxicity of NK-92 cells against DU145 cells.

### 3.6. Combined Treatment with Stattic, Tocilizumab, and Anti-TIGIT Increases Cytotoxicity in NK-92 Cell Coculture with DU145 Cells

**Figure 6:**
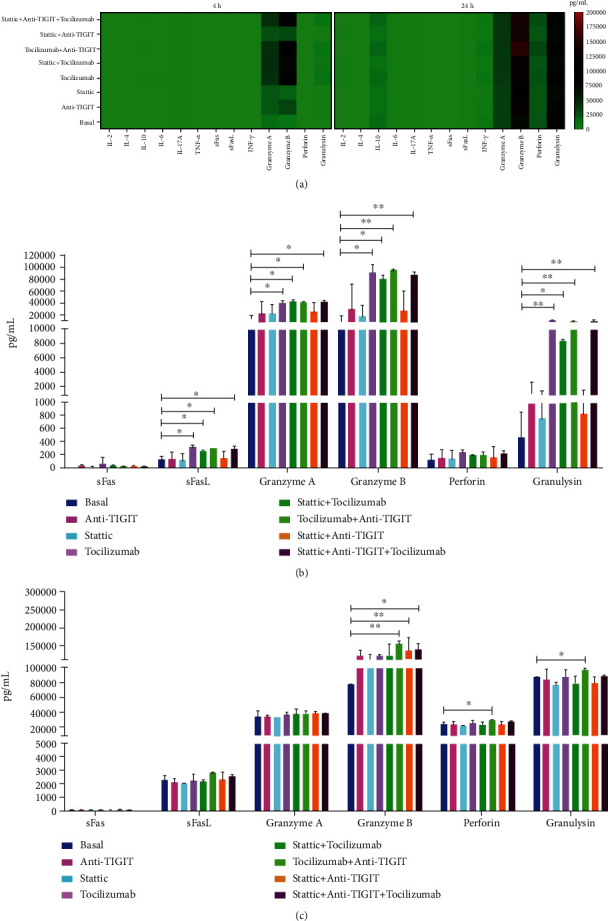
NK-92 cells increase the secretion of sFasL, granzyme A, granzyme B, and granulysin after treatment with stattic + Anti-TIGIT + Tocilizumab. (a) Evaluation of the levels of 13 soluble molecules related to the cytotoxic activity of supernatants from cocultures was carried out for 4 and 24 h. (b) Increased secretion of soluble FasL, granzyme A, granzyme B, and granulysin in the treated groups compared to the basal group in the coculture supernatant at 4 h. (c) The production of granzyme B, perforin, and granulysin is significantly maintained for 24 h in the treated groups. Data are shown as the mean ± SD. ∗*p* < 0.05, ∗∗*p* < 0.01, ∗∗∗*p* < 0.001 (unpaired *t*-test).

We evaluated molecules related to the cytotoxic activity of NK cells (Figure 6(a)). In comparison with the basal group, we found a significant increase in soluble FasL, granzyme A, granzyme B, and granulysin in the Tcz + anti-TIGIT and Stt + Tcz + anti-TIGIT groups at 4 h of coculture (Figure 6(b), ∗∗*p <0.01*). Moreover, when evaluating the production of these molecules at 24 h, it was observed that the production of granzyme B remained increased mainly in the same groups. Moreover, the production of perforin and granulysin remained significantly increased in the group of Tcz + anti-TIGIT (Figure 6(c), ∗∗*p* < 0.01). Our results show that the different treatments increased the secretion of FasL, granzyme A, granzyme B, and granulysin in the cocultures. Also, the combined use of Stt, Tcz, and anti-TIGIT increased the functional capacity of NK cells against DU145 castration-resistant prostate cancer cells.

## 4. Discussion

The immune response to prostate cancer, particularly in CRPC, has been characterized by poor cytotoxic activity; additionally, an immunosuppressive tumor microenvironment is often reported [31]. Our study found that cell models of advanced prostate cancer demonstrated constitutive expression of STAT-3, and also exhibited characteristics such as a high secretion of growth factors, such as VEGF and cytokines, such as IL-6 and CXCL8. These factors are closely related to the activation of this transcription factor and regulation of the immune response mediated by NK cells. For instance, studies have reported that constitutive activation of STAT-3 is an essential mediator for the secretion of VEGF, CXCL8, and IL-6, creating autocrine feedback (when these molecules bind their receptors, increased STAT-3 signaling is observed) [32, 33]. Additionally, the presence of TGF-*β* contributes to the activation of STAT-3 through phosphorylation of SHP-2 and is an important regulator of immune responses in this pathology [34, 35]. Furthermore, understanding the complex role of the tumor microenvironment is vital in order to propose and develope new treatment approaches in these cancers; this is mainly because some tumor environment-secreted factors, such as VEGF and TGF-*β*, are important regulators of therapeutic antibody blocking efficiency at certain immune checkpoints [36]. Simultaneously, comprehending all this is crucial, due mainly to the microenvironment's repercussions directly affecting NK cell cytotoxicity and functional capacity in this type of tumor [37–40]. Ultimately, recent research has reported that the presence of IL-6 and CXCL8 modulates the activity of NK cells by downregulating the expression of activating receptors and impairs the functional cytotoxic activity of these cells [41],and can induce the polarization of NK cells into an angiogenic phenotype, which also has been associated with the recruitment and polarization of macrophages to an M2 phenotype [42].

Likewise, the expression of ligands that limit the activity of NK cells in the microenvironment also suffers alterations during tumor progression. Thus, we mainly found that CD155, a receptor belonging to the PVR family and the principal ligand of the TIGIT immunoreceptor, is increased in the metastatic CRPC cell line. Previously, some studies have reported the implications of this ligand in the tumor microenvironment as it is directly involved with different factors, which are usually related to decreased patient survival [15, 43, 44]. Therefore, CD155 has been proposed as a potential therapeutic target in this disease [22]. Furthermore, some studies have observed that expression of CD155 could be induced by pathways such as Raf-MEK-ERK-AP-1 and through the Sonic Hedgehog pathway, which is highly active in advanced prostate cancer and can be indirectly mediated by STAT-3 via regulation of the TTF-1 promoter [44–46]. For this reason, it is essential to observe the pattern of CD155 expression in this pathology due to its implications for the regulation of immune checkpoint blockade through its combined expression with other ligands such as PDL-1 [47]. This combined expression of CD155 and other checkpoint ligands may prove to be an important variable that might help predict TIGIT blockade efficacy and thus enable the suitable selection of patients with advanced prostate cancer most likely to benefit from such a treatment.

Studies have reported that the tumor cells with constitutive expression of STAT-3 maintain the activity of this pathway through positive autocrine feedback between IL-6 and the IL6R [48, 49]. We observed by Western blot that the castration-resistant DU145 cells that maintain active STAT-3 exhibited a higher expression of the IL6R compared to the other cell lines; In this sense, some studies have observed, particularly in breast cancer, that constitutive expression of STAT-3 is increased in the tumor tissue and is even associated with risk factors such as mammographic density, which is why it has been proposed as a prognostic factor for determining risk in patients [50]. This highlights the feasibility of generating a therapy directed against the IL6R/STAT-3 axis in advanced stages of prostate cancer with these characteristics. We also evaluated the inhibition of this entire axis. We observed a reduction in metabolic activity and the dephosphorylation of STAT-3 when both inhibitors were administered. This effect was observed using lower concentrations of Stt than needed when Stt alone was used; here, the anti-IL6R seems to be sensitizing these tumor cells. In addition, various groups have described the effects of Stt (alone and/or in combination with other drugs) on the proliferative capacity, reduction of metastasis, and sensitization of tumor cells to drugs or immune responses in both *in vitro* and *in vivo* studies [51–55]. For example, it has been reported that inhibition of the IL-6-JAK/STAT-3 signaling pathway in CRPC cells (C4-2 and CWR22Rv1) sensitizes tumor cells to NK cell-mediated cytotoxic action through changes in the PDL-1 ligand interaction and the expression of NKG2D [56]. Additionally, tocilizumab has already been used in some cancer models with important effects in different tumor cell models, mainly on the proliferation, invasion, and sensitization to drugs through dephosphorylation of STAT-3 [29, 57–59]. Recently, tocilizumab has been proposed as a component of combination therapy with other targeted monoclonal antibodies against immune checkpoints in phase I/II studies against multiple myeloma (NCT04910568) and melanoma (NCT03999749); interestingly, there is no such combined therapy targeted directly to prostate cancer as of yet. However, a recent study confirmed that combined inhibition of the JAK1-2/STAT-3 pathway and PD-L1 blockade decreases the escape of CRPC cells from the cytotoxic action of NK cells under hypoxic conditions [60], indicating the importance of implementing STAT-3 inhibitors in combination with blockers directed against highly expressed targets in prostate cancer.

Other research has shown the implications of STAT-3 on the immune response of NK cells, mainly on the regulation of cytotoxic mechanisms and on the expression of activation receptors [12]. For this reason, we decided to evaluate the effects of the treatments on these immune response cells. Interestingly, we observed that the use of stattic and tocilizumab in combination increases the expression of NKp46 in NK-92 cells. This activating receptor has previously been reported as an essential mediator of the immune response of cytotoxic cells in prostate cancer [8]. However, NKp46 can be regulated by STAT-3 mainly because this transcription factor can bind to promoter regions of the gene for this activating receptor [61]. So, we propose that using these treatments can avoid this regulation. Likewise, we observed a decrease in the expression of TIGIT, which has not been previously reported, and which allows us to hypothesize a direct interaction between the IL6R/STAT-3 axis and the regulation of this inhibitory receptor.

Based on the above, we evaluated the response of NK-92 cells against DU145 castration-resistant prostate cancer cells using the xCELLigence system, which allows a more accurate and robust observation of the efficacy of treatments and the response of cytotoxic cells, BiTES, and CAR-T cells in *in vitro* studies [28]. Additionally, the use of the xCELLigenceplatform has allowed us to accurately observe the effect of both treatments and the cytotoxic activity of the NK cells from short times to prolonged times of activity, which allows us to predict more accurately the possible behavior of these treatments in future *in vivo* studies.

Using the xCELLigence plates, we observed that combined therapy against the IL6R/STAT-3 axis and TIGIT increased the cytotoxicity of NK cells against tumor cells. We observed this increased cytotoxicity beginning from the first hours of coculture and continuing through the full 24 h of the experiment. In agreement with our results, some trials have demonstrated an enhanced NK cytotoxic response after the silencing of IL-6 in prostate cancer cells [56]. Likewise, other studies have observed positive effects on the cytotoxicity of NK cells after the inhibition of STAT-3 and the use of monoclonal antibodies directed against immune checkpoints [52, 62]. For example, the blockade of TIGIT was found to positively regulate the response of NK cells against breast cancer cell lines [63]. Furthermore, another study proposed the importance of NK cells on the efficacy of TIGIT blockade by observing a preventive effect on exhaustion in NK cells, which increased the response of CD8^+^ cells against colon CT26 cancer cells [64]. Another group recently observed that blocking TIGIT allows the reconstitution of antitumor activity when used together with an inhibitor of HIF-1*α*, a critical mediator of the immune response, which in turn is regulated by STAT-3 [65, 66]. Together, the evidence denotes the importance of using these treatments directed against IL6R/STAT-3 and TIGIT in combination in future animal models and patient studies. We expect that the positive antitumor effect would be mainly due to the regulation of the NK-mediated response and enhancement of CD8^+^ cells.

When we evaluated the supernatant from the coculture groups, we observed a significant increase in the secretion of cytotoxic molecules, mainly in the group treated with all inhibitors in the first hours of activity. Additionally, the increased secretion was maintained significantly in some of the groups treated in combination, even at the longest time points of the experiment. Previous studies have observed that inhibitors against IL-6, the main activator of STAT-3, recover the production of granzyme B [41]; another research demonstrated that silencing of STAT-3 enhances the production of granzyme B and perforin in NK cells [67], so we propose that using tocilizumab, stattic, and anti-TIGIT in combination allows these mechanisms to be reconstituted to a greater degree than using only a single inhibitor of the IL6R/STAT-3 axis for a more prolonged time.

Understanding the roles of these receptors, ligands, and pathways is essential for hypothesizing the possible mechanisms mediating the NK cells' activity. Therefore, we propose that inhibition of the IL6R/STAT-3 axis allows the regeneration of natural mechanisms directly involved in the cytotoxic and functional capacity of NK cells via a decrease in the expression of TIGIT, an inhibitory receptor with a high affinity for ligands present in these tumor cells. Supporting this, some researchers have observed that NK cells with high expression of TIGIT have a low cytotoxic capacity, mainly due to their greater affinity for CD155 and that the TIGIT/CD155 binding avoids interaction with the activating receptor CD226 and prevents its homodimerization [68, 69]; thus, the use of a monoclonal antibody against TIGIT could avoid the interaction with this inhibitory ligand and improve the response against these tumor cells. Likewise, we determined that using these treatments in combination with anti-TIGIT increases and maintains the expression of some factors such as NKp46 and secretion of granzyme A, granzyme B, perforin, and granulysin which may regulate cytotoxicity against DU145 prostate cancer cells.

## 5. Conclusion

In conclusion, this is the first study to propose a potential combination therapy directed against the IL6R/STAT-3 axis and TIGIT. Targeting these three factors simultaneously in a model of advanced castration resistance prostate cancer revealed a significant improvement in the functional activity of NK cells against these tumor cells, which is determinant for the design of future studies or clinical trials examining the *in vivo* efficacy of these treatments.

## Data Availability

All the original contributions presented in this study are included in the article. Further inquiries can be directed to the corresponding authors.

## Supplementary Materials

Supplementary 1 Figure S1: Evaluation of treatment effects over non-neoplastic prostate cells. RWPE-1 cells were exposed to the treatments for 24 h. Subsequently, determination of viability (a) and metabolic activity (b) was performed. All experiments were repeated at least three times. Data are shown as the mean ± SD. ∗*p* < 0.05, ∗∗*p* < 0.01, ∗∗∗*p* < 0.001; (ANOVA with Bonferroni multiple comparison test).

Supplementary 2 Figure S2: Coculture experiments through the xCELLigence platform. (a, c) DU145 target cells were cultured for 19 h for their adhesion; in brief, the NK-92 cells were placed in ranges 5 : 1 and 10 : 1, the loss of impedance (cell index) was monitored in cocultures for 24 h. (b, d) The percentage of cytolysis was calculated from the obtained cell index at 4 h and 24 h.
