# Supplementary figures and images for: Combination Blockade of the IL6R/STAT-3 Axis with TIGIT and Its Impact on the Functional Activity of NK Cells against Prostate Cancer Cells
Source: J Immunol Res. 2022 Apr 12;2022:1810804. doi: 10.1155/2022/1810804 (PMC9020142; doi:10.1155/2022/1810804)

**a**

**RWPE-1**

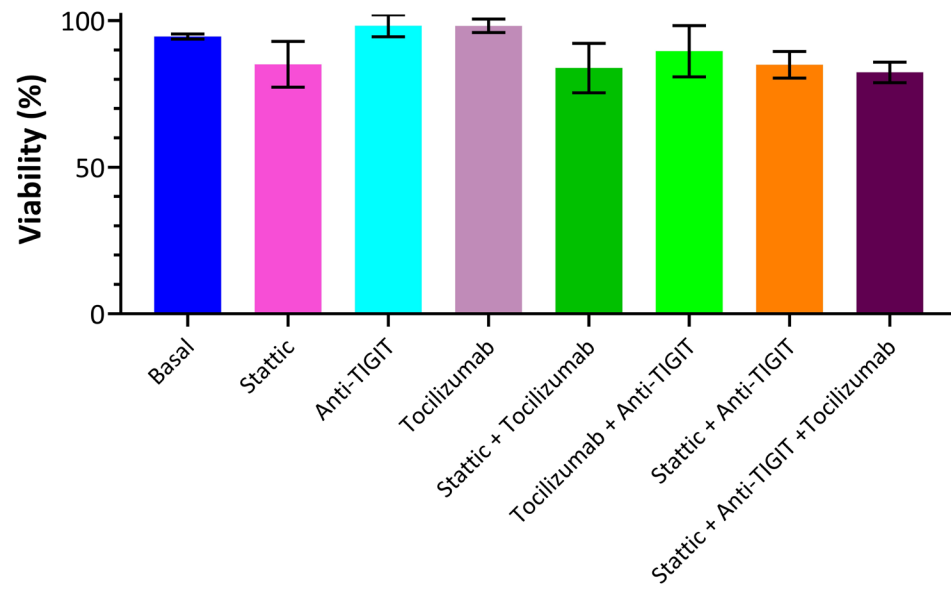

**b**

**RWPE-1**

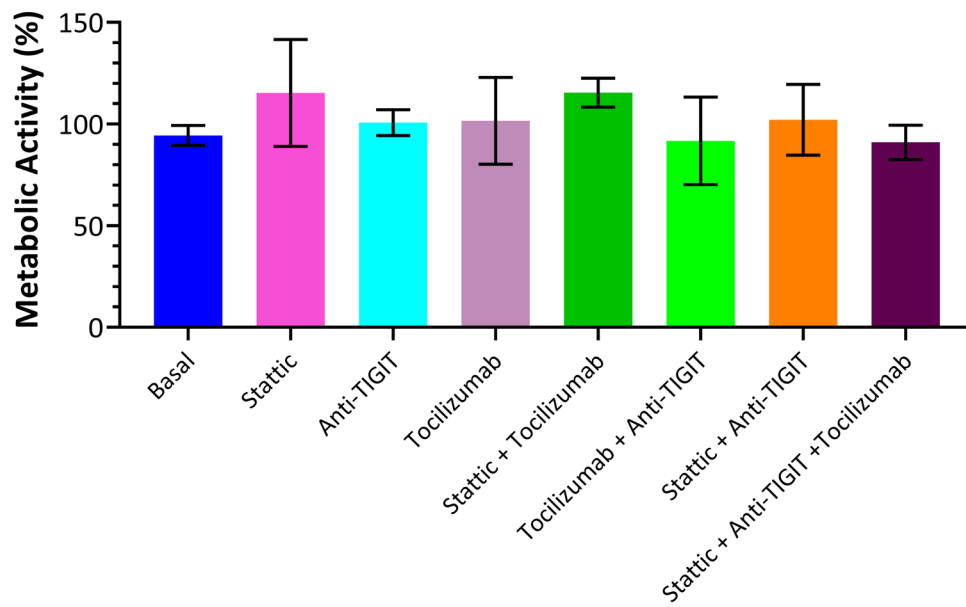

Supplement: Supplementary 1 — Figure S1: Evaluation of treatment effects over non-neoplastic prostate cells. RWPE-1 cells were exposed to the treatments for 24 h. Subsequently, determination of viability (a) and metabolic activity (b) was performed. All experiments were repeated at least three times. Data are shown as the mean ± SD. ∗p < 0.05, ∗∗p < 0.01, ∗∗∗p < 0.001; (ANOVA with Bonferroni multiple comparison test). [file 1810804.f1.pdf]

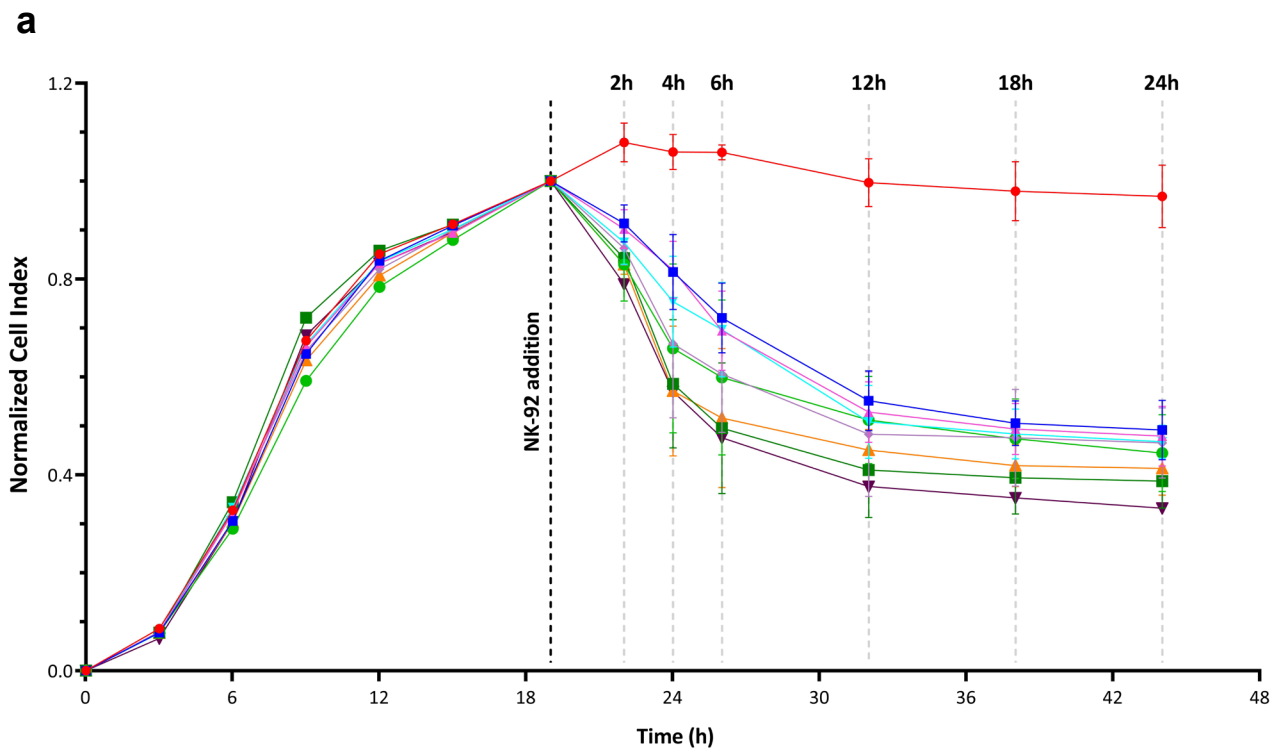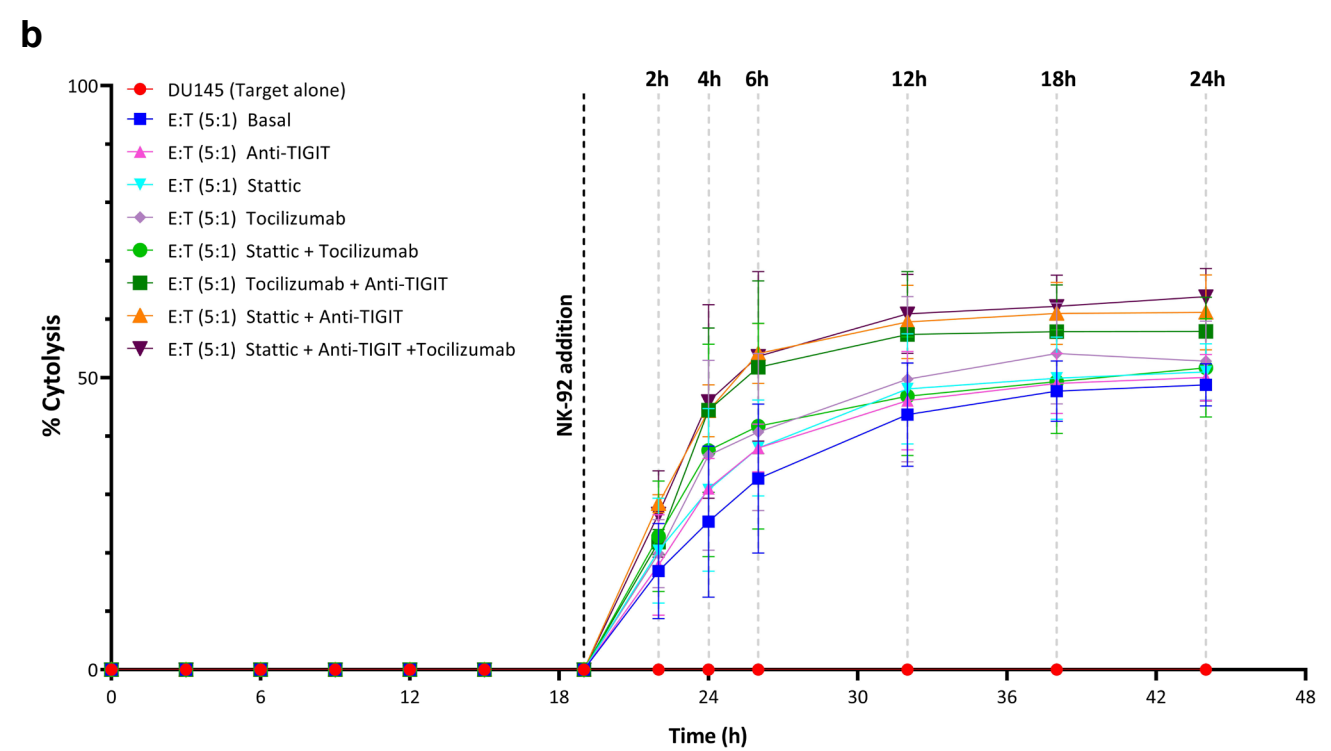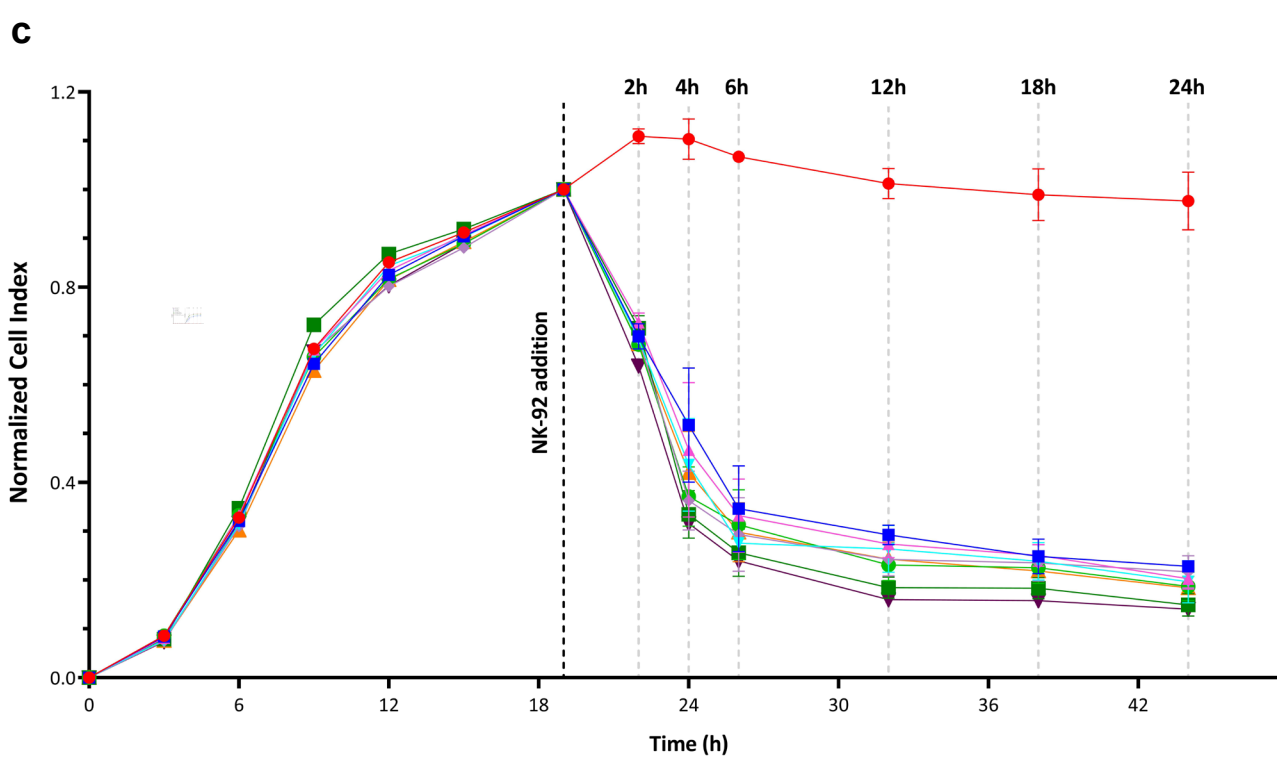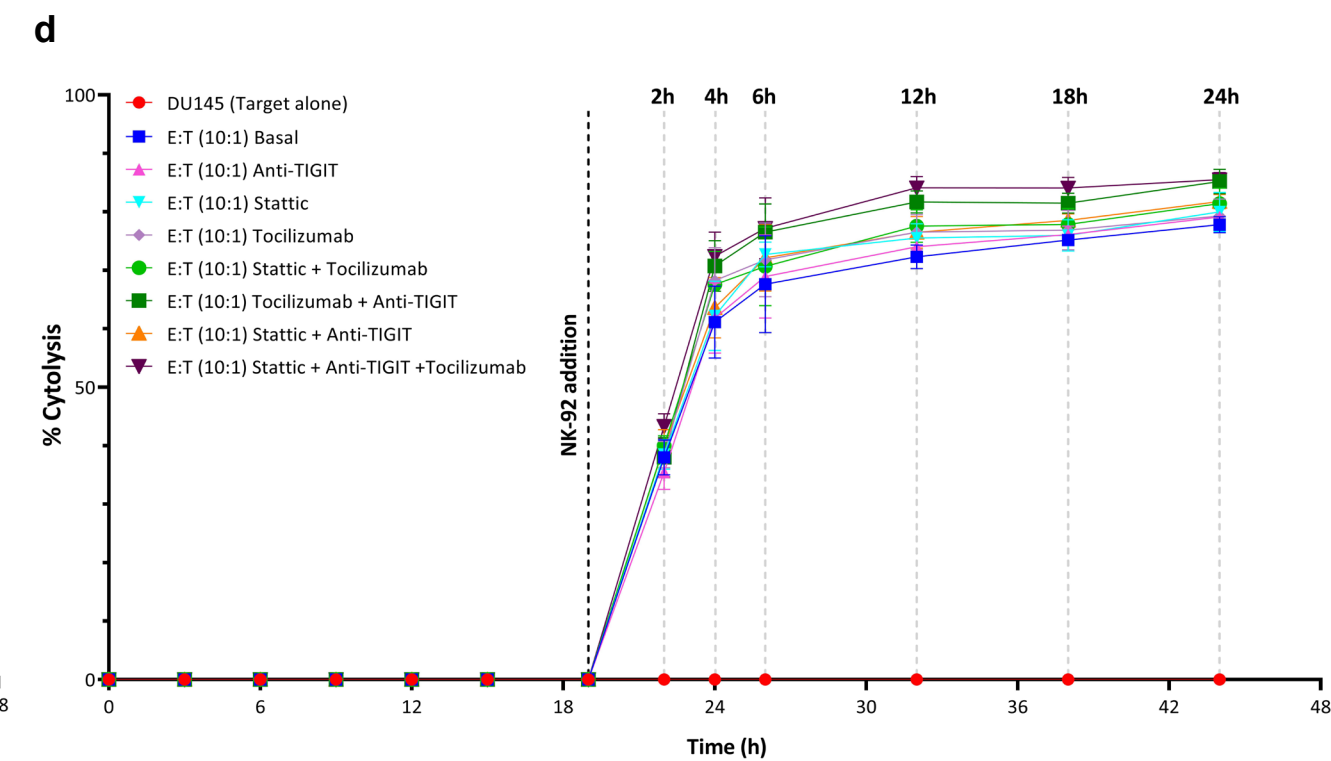

Supplement: Supplementary 2 — Figure S2: Coculture experiments through the xCELLigence platform. (a, c) DU145 target cells were cultured for 19 h for their adhesion; in brief, the NK-92 cells were placed in ranges 5 : 1 and 10 : 1, the loss of impedance (cell index) was monitored in cocultures for 24 h. (b, d) The percentage of cytolysis was calculated from the obtained cell index at 4 h and 24 h. [file 1810804.f2.pdf]
